# Yeast Smell Like What They Eat: Analysis of Volatile Organic Compounds of *Malassezia furfur* in Growth Media Supplemented with Different Lipids

**DOI:** 10.3390/molecules24030419

**Published:** 2019-01-24

**Authors:** Mabel Gonzalez, Adriana M. Celis, Marcela I. Guevara-Suarez, Jorge Molina, Chiara Carazzone

**Affiliations:** 1 Laboratory of Advanced Analytical Techniques in Natural Products (LATNAP), Universidad de los Andes, Cra 1 No. 18A-12, Bogotá 111711, Cundinamarca, Colombia; mabel.c.gonzalez@uniandes.edu.co; 2 Grupo de Investigación Celular y Molecular de Microorganismos Patógenos (CeMoP), Universidad de los Andes, Cra 1 No. 18A-12, Bogotá 111711, Cundinamarca, Colombia; mi.guevara34@uniandes.edu.co; 3 Laboratorio de Micología y Fitopatología (LAMFU), Universidad de los Andes, Cra 1 No. 18A-12, Bogotá 111711, Cundinamarca, Colombia; 4 Centro de Investigaciones en Microbiología y Parasitología Tropical (CIMPAT), Universidad de los Andes, Cra 1 No. 18A-12, Bogotá 111711, Cundinamarca, Colombia; jmolina@uniandes.edu.co

**Keywords:** fungal VOCs, GC-MS, HS-SPME, lipids, exponential growth phase, stationary growth phase, oleic acid, palmitic acid

## Abstract

*Malassezia furfur* is part of the human skin microbiota. Its volatile organic compounds (VOCs) possibly contribute to the characteristic odour in humans, as well as to microbiota interaction. The aim of this study was to investigate how the lipid composition of the liquid medium influences the production of VOCs. Growth was performed in four media: (1) mDixon, (2) oleic acid (OA), (3) oleic acid + palmitic acid (OA+PA), and (4) palmitic acid (PA). The profiles of the VOCs were characterized by HS-SPME/GC-MS in the exponential and stationary phases. A total number of 61 VOCs was found in *M. furfur*, among which alkanes, alcohols, ketones, and furanic compounds were the most abundant. Some compounds previously reported for *Malassezia* (γ-dodecalactone, 3-methylbutan-1-ol, and hexan-1-ol) were also found. Through our experiments, using univariate and multivariate unsupervised (Hierarchical Cluster Analysis (HCA) and Principal Component Analysis (PCA)) and supervised (Projection to Latent Structures Discriminant Analysis (PLS-DA)) statistical techniques, we have proven that each tested growth medium stimulates the production of a different volatiles profile in *M. furfur*. Carbon dioxide, hexan-1-ol, pentyl acetate, isomer5 of methyldecane, dimethyl sulphide, undec-5-ene, isomer2 of methylundecane, isomer1 of methyldecane, and 2-methyltetrahydrofuran were established as differentiating compounds among treatments by all the techniques. The significance of our findings deserves future research to investigate if certain volatile profiles could be related to the beneficial or pathogenic role of this yeast.

## 1. Introduction

All species in the genus *Malassezia* are lipid-dependent because of their inability to synthesize de novo C_14_ or C_16_ fatty acids (FAs) [1] due to the lack of a cytosolic fatty acid synthase complex (FAS) [2]. In order to survive, *Malassezia* rely on lipids encountered in host skin that are subsequently metabolized by lipases and phospholipases [3]. The 18 species of the genus belong to class Malasseziomycetes of phylum *Basidiomycota* [4]. *Malassezia* species could represent between 53% and 80% of human skin fungal microbiota, accumulating at different body sites, especially behind the ears [5] and on the scalp [6].

These organisms have been recognized as aetiological agents of pityriasis versicolor and might also be related to other skin diseases, such as seborrheic dermatitis, dandruff, folliculitis, atopic/eczema dermatitis syndrome, and psoriasis [7]. Depending on the stimulation, or occasionally the downregulation of the immune response against *Malassezia*, these yeasts could remain as a commensal or eventually become pathogenic [8]. The causes related to this interconversion are still unknown. There are different studies suggesting that a relationship exists between pathogenicity and modifications in the lipid compositions of the host [9,10] or changes in the skin microbiota [11,12]. The complexity of the human skin environment hinders the possibility of fully comprehending the host–microbe and microbe–microbe interactions [13], because the skin determines the microbiota and, at the same time, the resident microbial communities modify this environment in a dynamic process [14]. Host demographics and genetics, human behavior, local and regional environmental characteristics, and transmission events determine the human skin microbiota variability [15]. The spatial distribution of the micro-organisms is also highly variable [5]. *Malassezia* and *Cutibacterium acnes* (formerly *Propionibacterium acnes*) [16] reside in areas with a high density of sebaceous glands, whereas *Staphylococcus* and *Corynebacterium* are mostly prevalent in areas with a high temperature and humidity [17]. Any changes in these conditions may affect homeostasis and promote a pathogen or even a commensal, such as *Malassezia*, to cause disease.

Some *Malassezia* species can easily be differentiated by the types of lipids necessary to grow them in certain media [18,19]. Therefore, the *Malassezia* assimilation assay is widely used to determine the lipidic requirement of different strains [3]. For example, palmitic acid (PA) has a fungicidal activity in *Malassezia pachydermatis*, in an atypical strain of *Malassezia furfur*, and in *Malassezia sympodialis*, but not in *M. furfur* CBS 1878 [20]. The addition of oleic acid (OA) produces a fungistatic effect on *M. pachydermatis*, on atypical *M. furfur*, and on *M. furfur* CBS 1878, but on *M. sympodialis* OA produces a fungicidal effect [21]. Therefore, Dixon, mDixon, and Leeming and Notham (LNA) media are the most widely used for culturing *Malassezia*, because their more complex composition and the presence of Ox bile (obtained after purifying and drying fresh bile from oxen) offer the essential nutritional requirements for their growth [8,22]. The ability of *M. furfur* to survive in PA could not be related to the presence of a Δ9-desaturase gene OLE1, which is involved in the conversion of saturated to unsaturated acids, such as PA to palmitoleic acid, because this gene has also been found in other species/strains where PA is fungicidal [3,21].

Thus, the lipid-dependent and lipophilic lifestyle of *Malassezia* spp. could determine its ecological niche in the skin environment of human and animal hosts [20]. Understanding new aspects of the lipid metabolism of these yeasts is expected to provide new insights into their biology and ecology, and, therefore, illuminate the path to understanding interconversions between commensalism and pathogenicity [21]. As they can be easily collected, fungal volatile organic compounds (VOCs) are one of the means to assess the metabolic profiles of fungi related to certain diseases [23,24,25,26,27,28,29]. Furthermore, they can be used to relatively quickly monitor static or dynamic metabolic changes [27,30,31,32]. Other important uses of fungal VOCs include biotechnological processes [30,33,34], signalling of ecological communication between different species [31,35], and obtaining information about fungal development [36,37]. The high diversity of VOCs released by bacteria and fungi justifies the creation of a database that summarizes the taxonomic distribution of around 2000 compounds and their possible functions. The first version of the mVOC Database was created in 2014, and was renewed this year [38,39]. No volatile compounds of *Malassezia* have been reported in this database, because the data sources used have been limited to PubMed publications [39]. The first and only description of *Malassezia* volatiles, in which the authors reported the presence of 11 different VOCs, was performed in 1979, when the genus was recognized as *Pityrosporum* [40].

The human skin sebum contains a complex mixture of triglycerides, fatty acids, wax esters, sterol esters, cholesterol, and squalene. Among the fatty acids, the unsaturated ones predominate, but the sebum is generally composed of OA, PA, stearic acid, linoleic acid, sapienic acid, and palmitoleic acid [41]. OA is an irritant component that can induce dandruff in dandruff-susceptible people [10,41]. Among animals, PA is a fatty acid found in the human sebum in a relatively high amount [21]. Having this in mind, the aim of this study is to investigate how the lipid composition of a liquid medium influences the production of VOCs released by *M. furfur*, the only *Malassezia* species known to survive in the PA medium. To accomplish this, four different media for growing the strain CBS 1878 were prepared: (1) mDixon, (2) minimal medium (MM) supplemented with oleic acid (OA), (3) MM with oleic + palmitic acid (OA+PA), and (4) MM with palmitic acid (PA). The VOCs released by the yeast grown in these media were analyzed using headspace solid-phase microextraction (HS-SPME) and gas chromatography-mass spectrometry (GC-MS). The mDixon medium was selected as the reference for in vitro growing conditions. The other media serve to identify the VOCs released under restricted nutritional conditions when an unsaturated acid (OA), a saturated acid (PA), or a combination of both is present in the medium. In addition, samplings of VOCs were performed for each medium in the exponential and stationary phase. Finally, results from eight experimental treatments (mDixon in both growing phases: De, Ds; oleic acid in both growing phases: Oe, Os; oleic + palmitic acid in both growing phases: OPe, OPs; and, finally, palmitic acid in both growing phases: Pe, Ps) were obtained.

Previous reports on other species have demonstrated that changes in the microbial growth conditions produce qualitative and quantitative alterations in the VOCs released by an organism [28,42,43]. Based on these findings, we hypothesized the existence of a chemical differentiation in the VOCs profiles released by *M. furfur* CBS 1878 due to the different lipid composition of the medium and to the phase of growth. Univariate comparisons and unsupervised and supervised multivariate analyses have been applied to measure the metabolic differentiation and its significance.

## 2. Results and Discussion

### 2.1. Profiling of VOCs from M. furfur

**Figure 1:**
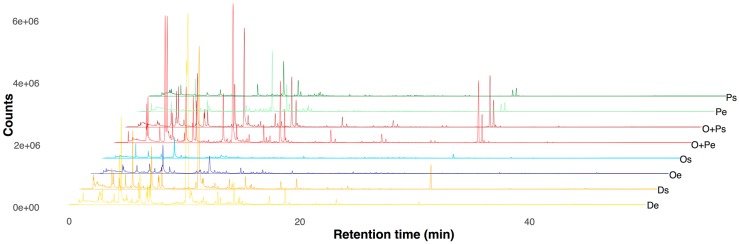
Chromatograms of *Malassezia furfur* in exponential and stationary phases of growth and in eight treatments with different fatty acid (FA) content. De = Dixon in Exponential phase, Ds = Dixon in Stationary phase, Oe = Oleic acid in Exponential phase, Os = Oleic acid in Stationary phase, OPe = Oleic acid + Palmitic acid in Exponential phase, OPs = Oleic acid + Palmitic acid in Stationary phase, Pe = Palmitic acid in Exponential phase, Ps = Palmitic acid in Stationary phase. The Y-axis denotes chromatographic intensity of signals in an absolute scale after being detected in the mass spectrometer.

HS-SPME/CG-MS analysis was applied to determine the profiles of VOCs released by *M. furfur* CBS 1878 growing in four different media: mDixon, OA, OA+PA, and PA in both growing phases. With the objective of analyzing exclusively VOCs released by the organism, the following criteria were established for the inclusion of a compound originating from the micro-organisms in the succeeding matrices: (1) the VOC was absent in any replicate of all medium control analyses (for more details, see the Materials and Methods section); and (2) the VOC was found in more than one replicate of the yeast. Qualitative differences between the chromatographic profiles of the eight experimental treatments (Figure 1) show a combination of both: the VOCs released by *M. furfur* and the VOCs released from growth media.

HS-SPME/CG-MS analysis of *M. furfur* allowed for the annotation of 61 different VOCs (Table 1), among which alkanes, alcohols, ketones, and furanic compounds were the most prevalent. Carbon dioxide, hexan-1-ol, octane, and nonane showed the highest peak areas. The lack of terpenes, which are common VOCs found in fungi [44,45,46], such as *Candida albicans* [44] and *Aspergillus fumigatus* [47], is remarkable. Average peak areas, standard deviations, and the number of replicates in which each VOC was found are summarized in Table 1. A tentative annotation of 27 compounds was carried out by comparison of mass spectra with databases and experimental retention index (RI) deviations below 30 units from the theoretical RI. In opposition, 30 compounds were annotated to specific moieties, or at least to a chemical class, based only on mass spectra comparisons. Among these, most of the alkanes could not be exhaustively identified because their electron ionization (EI) fragmentation patterns are very similar. Four compounds were described as unknowns.

The presence of γ-dodecalactone, 3-methylbutan-1-ol, and hexan-1-ol, previously described as signature VOCs of *Malassezia*, was reported [40]. The γ-dodecalactone was detected in the mDixon and the OA medium in both growth phases, while the hexan-1-ol was found in all treatments, with the exception of the PA medium. In contrast to the first analysis of Labows et al. (1979), who found many different gamma lactones in the volatiles profile of *Malassezia*, we detected the presence of only one of these gamma lactones. These differences could be related firstly to the genetic differences among the tested strains, because, in 1979, they used the strains ATCC 24,047, 12,078, and 14,521 of *M. furfur* [40]; secondly, to the growing conditions, because the authors used solid media and Tween 80; or, finally, to the sampling conditions, because they absorbed the compounds in tenax, instead of using SPME (solid-phase microextracion) fibers [40]. Although mDixon also contains OA, the dietary importance of this unsaturated acid in the biosynthesis of γ-dodecalactone in *M. furfur* remains unknown. If the presence of OA controlled this pathway, the emission of γ-dodecalactone would have been observable also in the OA+PA medium, unless PA was an inhibitory supplement. In other fungi, OA is indeed a suitable substrate for this biosynthesis, undergoing hydroxylation, β-oxidation, isomerization, and lactonization reactions [33]. Taking into account the rest of the VOCs, it is clear that the DVB/CAR/PDMS-SPME fiber allowed for the sampling of a wider range of polarities than the ones analysed by Labows et al. using tenax in 1979. This resulted in the description of VOCs not previously reported for *Malassezia* and even for fungi, according to the data summarized in the mVOC 2.0 Database. The 16 compounds previously reported in other fungi are marked with * in Table 1.

From all of the volatiles profiles, 25 compounds were detected in the five replicates of all media, but the absence/presence of most of the compounds was highly variable. The maximum number of VOCs (27) was reported for Ds, followed by 23 compounds detected in Oe. Subsequently, 20 compounds were encountered in OPe, and, finally, just eight VOCs were revealed in Ps. PA medium produced a drastic reduction in the number of VOCs and even in the chemiodiversity, limited almost exclusively to alkanes. The comparison between the VOCs obtained from *M. furfur* CBS 1878 in the four media discussed, and the ones released by the strains ATCC 24,047, 12,078, and 14,521 of *M. furfur*, *M. pachydermatis*, and *Malassezia globose* grown in different media [40,48], demonstrates the importance of the lipid substrate for both the growing of micro-organisms and the releasing of VOCs. The interaction between the two variables growth and diversity of volatiles in *M. furfur* is still unknown because growing (determined by the CFU (colony forming units)) and volatile sampling (through HS-SPME/GC-MS) could not be performed simultaneously on each replicate in order to maintain the headspace conditions without air contamination. Anyways, we were able to collect indirect evidence that the lack of growth (the static patterns observed for the growth curves in PA and OA+PA media in Figure S1) does not correlate with the quantity of released VOCs. While the number of detected VOCs shows that PA more greatly reduces the diversity of VOCs, in the other growth-static medium (OA+PA) *M. furfur* released more than 15 volatiles (Table 1). 

### 2.2. VOCs Profile Differentiation in Four Growth Media in the Exponential and Stationary Phase

Unpublished data by Celis et al. (2017) showed that *M. furfur* CBS 1878 is capable of growing in modified Dixon (mDixon) and OA media, whereas it can only survive in OA+PA and PA media (Figure S1), as can be verified by the absence of growth (exponential phase) in these last two media [21]. When the growth medium is supplemented with a mixture of PA and OA, the toxic effect of PA is, at least partly, relieved and *M. furfur* is able to grow (Figure S1). In this research, using a univariate (UVA) and a multivariate (MVA) statistical analysis, we proved that the growth media and phases produced differences in the volatile profiles released by *M. furfur*.

**Figure 2:**
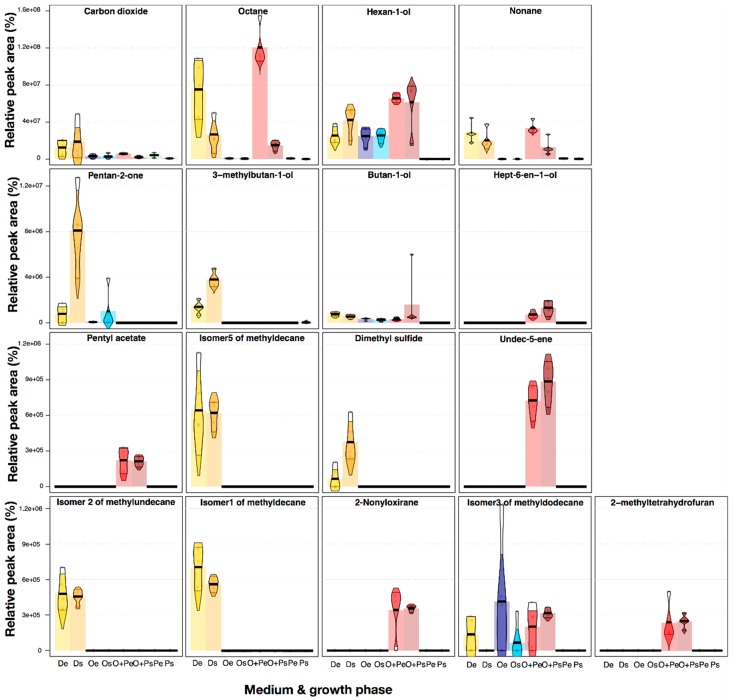
Pirate-plots representing univariate statistical differences in the absolute peak areas of 17 VOCs in which the grouping variable Medium-Phase significantly affects the release (*P* < 0.05 in ANOVA and a Kruskal—Wallis test). Variation of data is represented by Bayesian High-Density Intervals after running 10,000 iterations. The bars show the median values for each treatment. The first line of plots is y-scaled from 0 to 16 × 10^7^; in the following line, the y-axis was scaled from 0 to 1.2 × 10^7^, while the other two lines are scaled from 0 to 1.2 × 10^6^.

We demonstrated that, for 17 VOCs, peak areas significantly change when the medium-growth phase is modified (ANOVA, 2.37 < F < 560.26, *P* < 0.05) (Kruskal–Wallis test, 14.34 < K < 34.60, *P* < 0.05) (Table S1). We reported VOCs with *P* < 0.05 in a parametric and a non-parametric test, because more than half of the compounds (34 of 61) did not have a normal distribution (Shapiro–Wilk test, 0.59 < W < 0.91, *P* < 0.05). Figure 2 summarizes the semi-quantitative differences in the total peak areas between each treatment for these 17 VOCs. The peak areas of carbon dioxide released by *M. furfur* growing in mDixon were higher than in the other treatments, evidencing an increased respiration in this medium due to an increase in growth (Figure S1). Octane and nonane were released in the mDixon and OA+PA media with semi-quantitative differences in their areas. Hexan-1-ol, butan-1-ol, and the Isomer3 of methyldodecane were released in all media, except in PA. In contrast, the rest of the VOCs were released by *M. furfur* almost exclusively in the mDixon or in the OA+PA media (Figure 2). Differences among exponential and stationary phases were also observed, being some compounds increased in the stationary phase, such as dimethyl sulphide, and others decreased, such as octane. The presence of dimethyl sulphide, and other volatile organic sulphur compounds (VOSC), has been previously reported in bacteria [49], ascomycota yeasts [50,51], and basidiomycota yeast [52]; however, to the best of our knowledge, this is the first report of this compound in the genus *Malassezia*. Another commensal microfungi, *Candida albicans*, shared the presence of dimethyl sulphide, 2-methylpropan-1-ol, pentan-2-one, and 3-methylbutan-1-ol with *M. furfur* [53]; however, the ecological functions of these VOCs for both remain unknown. The presence/absence of some specific VOCs in *M. furfur* highlights the importance of analyzing the different volatiles profiles of a single species in different environments, especially when the objective of the research is to track biomarker compounds for chemotaxonomic, biotechnological [31], or therapeutic purposes [46]. Chemical differentiation in the VOCs released should be studied whenever possible. Previous reports using UVA showed that the emission of some VOCs in other fungi, such as *Ceratocystis sp.*, *Lentinus lepideus*, *Aspergillus versicolor*, *Aspergillus fumigatus*, *Penicillium commune*, *Cladosporium cladosporioides*, *Paecilomyces variotii*, *Phialophora fastigiata Trichoderma spp.,* and *Kluyveromyces marxianus*, depends highly on the differences in the nutritional supplement of the growth medium [25,42,43,54,55,56].

**Figure 3:**
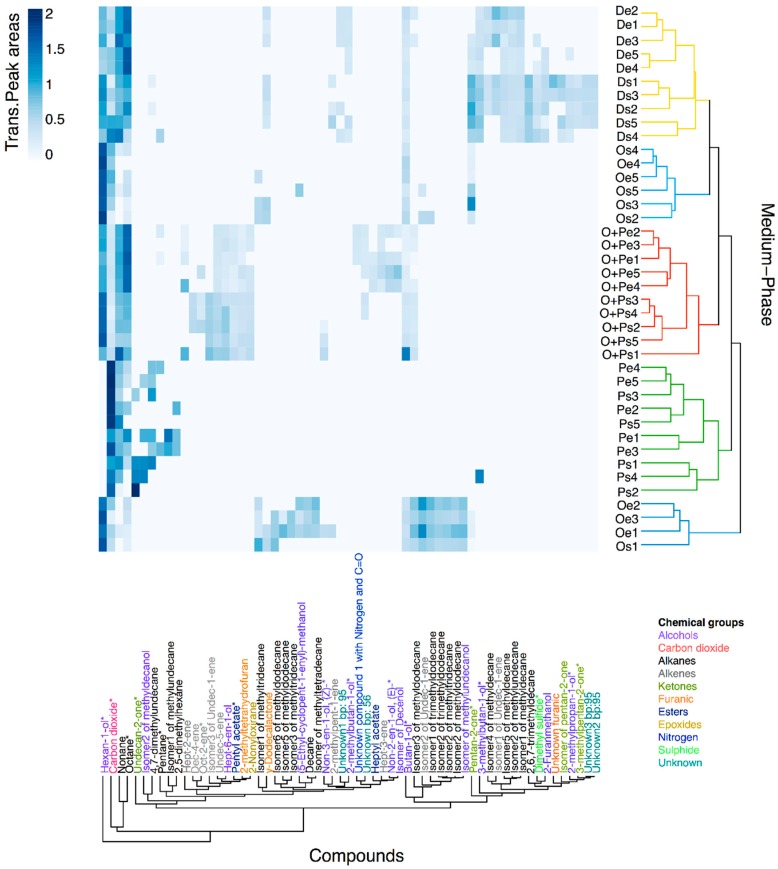
A heatmap of Hellinger transformed and Pareto-scaled peak areas from 61 tentative annotated volatiles found in an *M. furfur* analysis using HS-SPME/GC-MS. Dendrograms represent similarities between samples (objects) and VOCs (variables) using Euclidean distances as a measure. Clusters of medium-phase samples were coloured according to the four growth media, while the compounds were coloured according to the chemical group. Compounds with * represent VOCs reported previously to be released by other Fungi in the mVOC 2.0 Database.

Using Hierarchical Cluster Analysis (HCA), we discovered that the five replicates from *M. furfur* growing in the mDixon and OA+PA in the exponential and stationary phase have a signature profile that is easily differentiated from the profiles of other treatments; however, the grouping is not related to a specific chemical class of compounds (Figure 3). This aggrupation validates the reproducibility of the volatiles profiles, although some VOCs showed an evident high variance of transformed and scaled peak areas in the heatmap. In addition, the topology demonstrates that a dynamic transformation of VOCs production occurs when the yeast moves from the exponential to the stationary phase, even for the OA+PA medium in which growth remains static (Figure 3 and Figure S1). In the PA medium, higher semi-quantitative similarities between samples of different growth phases were found, which explain the lack of clustering of the five replicates of the exponential and stationary phase. Nevertheless, volatiles profiles from the PA medium are clustered together, demonstrating the differentiation among the VOCs. In contrast, the OA medium does not show a distinctive volatiles profile because of the higher variability between the VOCs found in each replicate in both growth phases. Some of these replicates only had a couple of VOCs with low-intensity peak areas and were clustered with the mDixon. The other group of replicates were clustered as the outgroup of the Dixon, OA, and OA+PA group, having more VOCs with higher peak areas. VOCs from the chemical groups detected in *M. furfur* can be related to main microbial biochemical pathways. Therefore, it can be suggested that furanic compounds, such as lactones, methylketones, esters, alkenes, and maybe alcohols, could be obtained through the catabolism of fatty acids; the alcohols also could be synthesized from pyruvate pathways intermediaries, alkanes probably could be obtained from poliketides generated in the acetate pathway, and sulphur and nitrogen compounds might be produced from amino acids [57,58].

**Figure 4:**
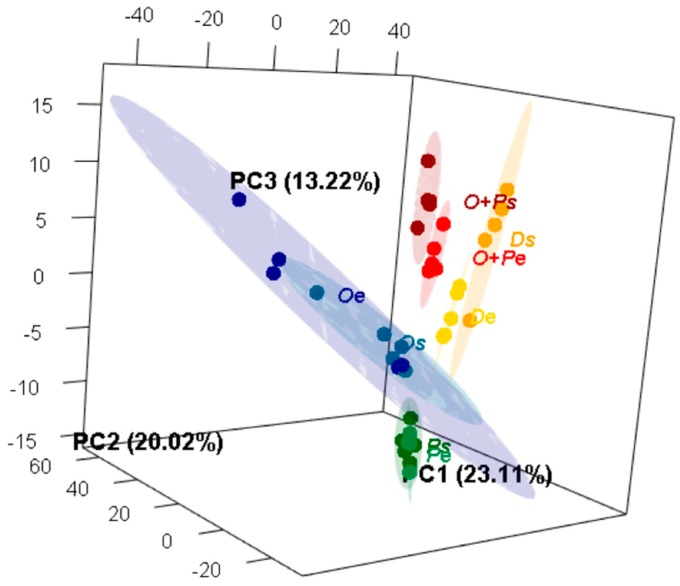
Three-dimensional Principal Component Analysis (PCA) of the volatiles profiles of *M. furfur* growing in eight different experimental treatments. The ellipses in this plot are confidence intervals of 95% using a normal distribution. For more details about the data distribution in the three axes, see the three-dimensional animation in Figure S2.

A second multivariate non-supervised approximation was performed using Principal Component Analysis (PCA) to summarize the total variation of the volatiles profiles of *M. furfur* in each medium and growth phase, reducing the redundancy between correlated VOCs (Figure 4 and Figure S2). The first three principal components (PCs) represented 56.35% of the explained variance from the 61 volatiles annotated in the original matrix. In comparison to the analysis obtained with the HCA, all media and medium-phases were differentiated in a three-dimensional space, including the samples obtained from the OA medium, which were grouped together in the PCA space, despite having the higher variation represented by bigger ellipses. In order to determine whether the medium or the growth phase or both were variables affecting the volatiles profiles, all three were considered as grouping variables in the PCA analysis. Taking into consideration the total multivariate volatiles profile, the differentiation is better explained through the interaction medium-phase (PERMANOVA, R = 0.6945, F = 10.393, *P* < 0.001). Furthermore, when considered separately as independent variables, medium (PERMANOVA, R = 0.5457, F = 14.415, *P* < 0.001) and phase (PERMANOVA, R = 0.0516, F = 2.068, *P* = 0.043) were both also statistically supported as volatiles profile differentiation factors. PC1, which discriminates samples from both mDixon treatments, is associated with an increment in dimethyl sulphide, 2-methylpropan-1-ol, isomer1 of methyldecane, isomer5 of methyldecane, and the isomer2 of methylundecane. PC2, which discriminates samples from the OA+PA medium in the lower values, is associated with an increment in the isomer2 of undec-1-ene, the isomer2 of methyldodecane, the isomer2 of trimethyldodecane, the isomer of methylundecanol, and the isomer2 of methyltridecane. PC3, which discriminates samples from PA medium in the lower values, is associated with an increment in 2-methyltetrahydrofuran, hexan-1-ol, pentyl acetate, and undec-5-ene and a decrease in carbon dioxide. Half of the aforementioned compounds (9) also stood out in the UVA (Table S1 and Figure 2), but the PCA allows for the interpretation of the direction of differentiation of each compound according to the loading values on each axis. To the best of our knowledge, many studies have used MVA to evaluate differentiation of the metabolites profiles in bacteria and fungi to discriminate species, even for chemotaxonomic purposes [59,60,61,62], but this is the first report where MVA analysis demonstrates fungi chemical differentiation in the VOCs profiles that is caused by changes in both medium or/and phase of growth. There are some examples where the effect of one of these variables on the volatiles profile has been tested in bacteria [63,64,65]; however, the studies in fungi are less frequent [66].

**Figure 5:**
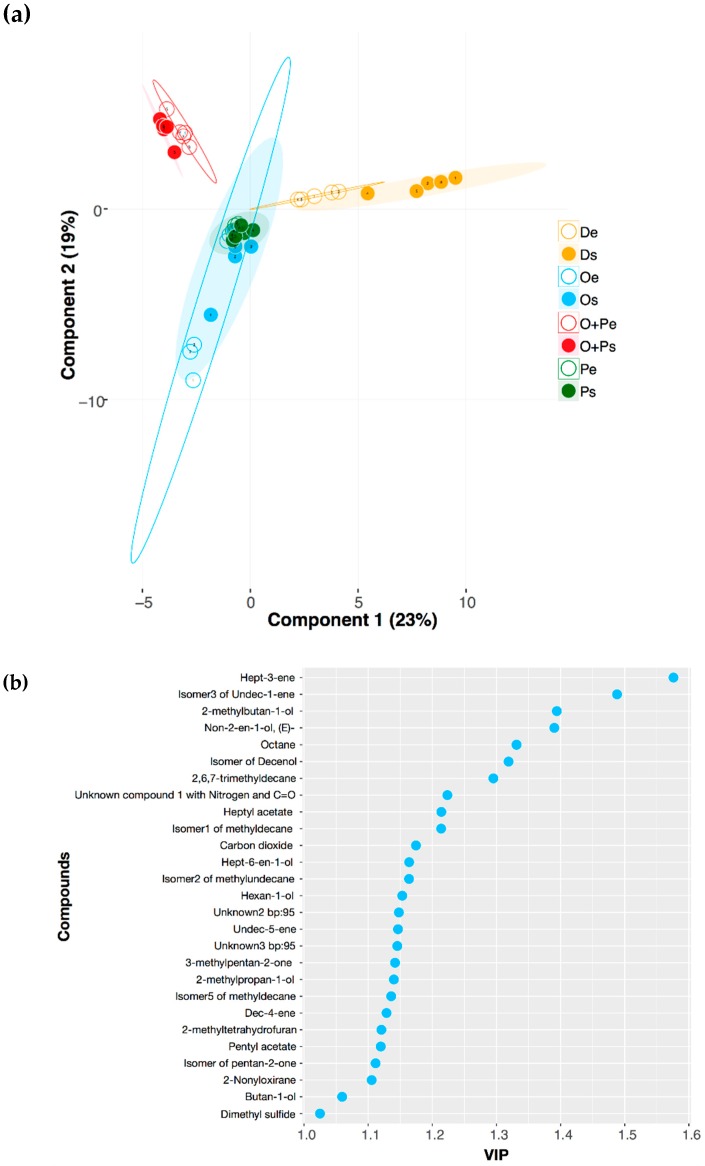
(**a**) Projection to Latent Structures Discriminant Analysis (PLS-DA) of volatiles profiles of *M. furfur* growing in eight different experimental treatments. The ellipses in this plot are confidence intervals of 95% using a *t*-distribution. (**b**) Variable importance on projection (VIP) plot showing the most important VOCs from *M. furfur* differentiated between four media and two growth phases. The VIP score of each compound was calculated as a weighted sum of the squared correlations between the PLS-DA components and the original variables.

A third multivariate supervised approximation was performed using Projection to Latent Structures Discriminant Analysis (PLS-DA, Figure 5a). The quality of the model was evaluated by the R2X, R2Y, and Q2 metrics. R2X and R2Y support the goodness of the fit, while Q2 > 0.5 reflects the predictability of the model as significant (R2X = 0.686, R2Y = 0.624, Q2 = 0.505). This analysis proves that the volatiles profiles from mDixon and OA+PA are differentiated, as well as the exponential and stationary phases of these two media. To ensure that a model based on a subset of the data can perform equally well on other subsets, we used cross-validation in seven groups and 1000 permutations. Despite the differences in the number of VOCs found between OA and PA media, as discussed before, Figure 5a shows a considerable overlap of these two media in both growth phases. This evidence suggests that some metabolic changes produced in *M. furfur* in both restricted dietary conditions could be similar, in comparison with the metabolic performance in OA+PA or mDixon. Our findings using this supervised technique demonstrate that the absence of both unsaturated oleic acid and saturated palmitic acid produces major changes in the fungal VOCs. The same was observed for the VOCs produced by *Saccharomyces cerevisiae* during the fermentation of different beverages, when linoleic acid is added at different concentrations [67]. The changes observed with *S. cerevisiae* deeply affect food quality in biotechnological and industrial processes, whereas, for *M. furfur*, these modifications help define the interactions between the yeast and other micro-organisms and the host. Surprisingly, the VOCs profile of the OA+PA medium is different from the sum of the ones obtained by the OA and PA media individually (Table 1, Figure 4 and Figure 5a). This has been previously observed in *Trichoderma spp*., where the volatiles profile found by combining three different amino acids in the growth medium was different from the sum of the profiles obtained when each amino acid was supplied individually [42].

The variable importance on projection (VIP) was used to assess the significance of the variables in the differentiation between treatments. Usually, values higher than 1 are considered significant. Twenty-seven VOCs from *M. furfur* followed this requirement (Figure 5b). Compounds found exclusively in one treatment or medium show the higher VIP values. For example, the hept-3-ene, which was found in the OPe treatment with a value of 1.58, is the compound that better differentiates the four media and the two growth phases, followed by isomer3 of undec-1-ene (VIP = 1.49), a signature compound from *M. furfur* growing in OPs. Among these 27 compounds, 12 were also signalled as statistically significant using UVA (Table S1 and Figure 2), findings that validate the interpretation of these two types of approximations. Nevertheless, the VIP values from PLS-DA offer more information because they discriminate the experimental treatments by organizing the VOCs in order of importance. On the other hand, this is not possible for the UVA, where each VOC is analyzed separately and not correlated to other compounds of the profile. Nine of the 27 VIP compounds had the higher loadings in the PCA analysis and were also highlighted as relevant in the UVA (carbon dioxide, hexan-1-ol, pentyl acetate, the isomer5 of methyldecane, dimethyl sulphide, undec-5-ene, isomer2 of methylundecane, isomer1 of methyldecane, and 2-methyltetrahydrofuran), whereas the PCA and PLS-DA analysis shared 10 out of these 27 VIP compounds. In addition to the abovementioned nine compounds, 2-methylpropan-1-ol was an important differentiation factor in both analyses. These findings evidence the differences of the multivariate models built under supervised and unsupervised conditions and the limitations of UVA. While the construction of the PCA model does not maximize distances between treatments, the PLS-DA constructs the multivariate components as a function of this response variable (in this case, the medium and phase of growth) [68]. This could explain why the VOCs with the higher VIP (for example, hept-3-ene), found exclusively in OPe, were not recognized as significant in the UVA or the PCA.

The ecological meaning of the differentiation produced by the absence of PA and OA on the volatiles profiles of *M. furfur* need to be further explored. PA is particularly abundant in human sebum, in comparison to other animals [21], so spatial differences in the PA distribution on skin could lead to spatial changes in the VOCs released by *M. furfur* or other species from human microbiota [10]. Therefore, a homeostasis needs to be established between human skin and microbiota through the production of metabolites. An enzyme of *Malassezia* that is able to attenuate the formation of biofilms of *Staphylococcus aureus*, an opportunistic bacterium from human skin microbiota [12], is already known; however, volatile metabolites released by these micro-organisms could have similar effects. VOCs functioning as quorum-sensing molecules [36,44] or antibiotics [58,69] have been reported in other bacteria and fungi; however, the ecological function of the VOCs released by *M. furfur* should be studied in the future. These studies show how any alteration in the human skin could be responsible for a disequilibrium in the homeostasis of the total microbiota and may be related to the interconversion of commensal to pathogenic states in *M. furfur*, as well as in other opportunistic species.

## 3. Materials and Methods

### 3.1. Chemicals

Chemicals were purchased from Sigma (St Louis, MO, USA) unless otherwise indicated.

### 3.2. Medium Preparation

Modified Dixon broth (mDixon), OA medium, OA+PA medium, and PA medium were prepared at the “Grupo de Investigación Celular de Microorganismos Patógenos (CeMoP)” at Universidad de los Andes (Bogotá, Colombia) one day previous to carrying out the experiments. The prepared mDixon contained 36 g/L malt extract (Oxoid), 10 g/L peptone (BD), 20 g/L Ox bile, 2 mL/L glycerol, 2 mL/L oleic acid, and 10 mL/L Tween 40 (Table S2). The OA, OA+PA, and PA media were prepared using a minimal medium (MM) that contained 10 mL K-buffer pH 7.0 (200 g/L of K_2_HPO_4_, 145 g/L of KH_2_PO_4_), 20 mL of M-N (30 g/L of MgSO_4_·7H_2_O, 15 g/L of NaCl), 1 mL 1% (*w*/*v*) of CaCl_2_·2H_2_O, 10 mL 20% (*w*/*v*) of glucose, 10 mL of 0.01% (*w*/*v*) FeSO_4_, 5 mL of spore elements (100 mg/L of ZnSO_4_·7H_2_O, 100 mg/L of CuSO_4_·5H_2_O, 100 mg/L of H_3_BO_3_, 100 mg/L of MnSO_4_·H_2_O, 100 mg/L of Na_2_MoO_4_·2H_2_O), and 2.5 mL 20% (*w*/*v*) of NH_4_NO_3_. A mixture of either 4.2 mM of palmitic acid (Merck, Darmstadt, Germany) or 4.2 mM of oleic acid (Carlo Erba), or 4.2 mM oleic acid and palmitic acid 50:50 (*v*/*v*), was added to the MM, respectively. Mixtures containing palmitic acid were supplemented with 1% Brij-58, an emulsifier that is not metabolized [20] (Table S3).

### 3.3. Yeast Growth

The strain CBS 1878 of *M. furfur* was purchased from the Fungal Biodiversity Center (Westerdijk institute, Utrecht, The Netherlands). Two loops of cells from 7-day-old mDixon agar grown colonies were suspended in 5 mL sterile water. Of these, 3 mL were added to 27 mL of mDixon broth or to 27 mL of MM with the respective fatty acid. After 3 days of growth at 180 rpm on a horizontal shaker (Heidolph, Germany), 0.3 mL culture were used to inoculate 29.7 mL of fresh mDixon broth or MM in 150 mL crimp-top Erlenmeyers with 20 mm PTFE septa (JG Finneran, Vineland, NJ, USA). The samples were incubated at 33 °C at 180 rpm. The reference time for the exponential phase was 1.17 days ± 0.17 and for the stationary phase was 3.08 ± 0.19 days. These times were previously validated as the mid-point of exponential phase growth and the initial stationary phase, respectively, in the mDixon (Figure S1) [21].

### 3.4. HS-SPME/GC-MS Analysis

Initially, VOCs released by each growth media alone were analyzed in three experimental replicates using the same 150 mL crimp-top Erlenmeyers, PTFE septa, under the same shaking and temperature conditions described for the growth of the yeast. These samples were used as a medium control analysis, with the aim of differentiating between the VOCs from each broth and those released by *M. furfur* CBS 1878 cultivated in such medium. Afterwards, VOCs released from *M. furfur* in both exponential and stationary phases and in the four liquid media: mDixon and MM supplemented with oleic acid (OA), or oleic and palmitic acid (OA+PA), or palmitic acid (PA), were analyzed in five replicates for each experimental treatment (De, Ds, Oe, Os, OPe, OPs, Pe, Ps).

In order to extract the widest range of polarities of VOCs, a SPME fiber of Divinylbenzene/Carboxen/Poly(dimethylsiloxane) (DVB/CAR/PDMS, 50 μm /30 μm, gray) (SUPELCO, PA, USA) was used. The fiber was exposed for 20 min to the headspace of the Erlenmeyer with growth media and yeast, maintained at 33 °C, and the agitation diminished to 90 rpm during the sampling, so that the state of the fiber was not compromised. Subsequently, the desorption process was carried out in the GC HP 6890 Series equipped with an Agilent Mass Selective Detector 5973 (Agilent technologies, Palo Alto, CA, USA) at 250 °C using splitless injection. Separation was performed on a BP-5 capillary GC column (30 m × 0.25 mm × 0.25 μm, SGE, Austin, TX, USA) using helium as a carrier gas at a flow rate of 1.3 mL/min. The temperature gradient program started at 40 °C for 0.5 min, followed by an increase to 60 °C at a rate of 6 °C/min, then the temperature was raised to 150 °C at 3 °C/min, and finally to 250 °C at 10 °C/min, and this temperature was maintained for 6 min. The GC-MS filament source and the quadrupole temperature were set at 230 and 150 °C, respectively. The electron ionization (EI) source was set at 70 eV, and the mass spectrometer was operated in full scan mode applying a mass range from *m*/*z* 30 to 300 at a scan rate of 2.0 scan/s. All samples, including linear alkanes, were run under the same chromatographic conditions. Linear alkanes of the series C_8_–C_20_ were used for the determination of retention indexes (RI) and later for the tentative assignment of compounds.

### 3.5. Data Analysis

To conduct the analysis of the GC-MS data, the profiles with VOCs obtained in five biological replicates for each medium-phase treatment were analyzed with the MSD ChemStation D.02.00.275 (Agilent technologies), and automatic integration using a threshold of 14 units was performed. Only those peaks absent in any of the three replicates of each medium control were considered to be volatiles released by *M. furfur*. Tentative annotation of compounds was done using NIST MS search 2.0 with the NIST 14 database and comparing experimental RI with the RI of compounds reported in the literature from the same (or an equivalent) column. Exported data files of chromatograms in .csv format were used for the subsequent plotting of chromatograms. Automatic integration results were carefully reviewed, and peak areas were used to construct the matrix in which tentatively annotated volatile compounds are reported as columns/variables and estimated peak areas as rows/observations. As grouping variables, we included the four media (mDixon, OA, OA+PA, and PA), both growth phases (exponential and stationary), and the combination medium-phase of the eight experimental treatments (De, Ds, Oe, Os, OPe, OPs, Pe, and Ps). Each peak area was transformed using a Hellinger transformation adequate for matrices with many zeros [70], and afterwards Pareto scaling was applied, which is useful for chromatographic analysis on a GC-MS platform [71].

In order to investigate the hypothesis that supervised techniques could afford more informative models than unsupervised analysis for the differential data, and thus unravel variables that influence class separation, each data set was analyzed by three techniques: Hierarchical Cluster Analysis (HCA), Principal Component Analysis (PCA), and Projection to Latent Structures Discriminant Analysis (PLS-DA). The data were scaled to have unit variance and were zero-centred. HCA is a statistical tool to pool samples based on similarities by measuring distances between all possible pairs of samples and plotting a topology for objects (e.g., samples for each treatment) and variables (e.g., VOCs). PCA was performed to reduce the redundancy between correlated VOCs. To support this analysis, PERMANOVA with 999 permutations was used using Euclidean distances in order to determine which grouping variable (medium, growth phase, or both) better explained the volatiles profiles differentiation. The PLS-DA model intended to find components or latent variables that correlate variation from a VOCs matrix to a response variable Y. The eight experimental medium-phase treatments (De, Ds, Oe, Os, OPe, OPs, Pe, Ps) were used as the Y response variable of the model. PLS-DA was cross-validated using seven groups (Q2 >0.5 was considered significant [72]) and performing 1000 random permutations of the VOCs dataset to evaluate the Q2 value. All statistical tests were performed using R studio software (http://www.rstudio.org/) and the stats packages HybridMTest (http://bioconductor.org/packages/HybridMTest/), vegan (https://CRAN.R-project.org/package=vegan), MetabolAnalyze (https://CRAN.R-project.org/package=MetabolAnalyze), psych (http://CRAN.R-project.org/package=psych), ropls (http://bioconductor.org/packages/ropls/) and ggplot2 (https://CRAN.R-project.org/package=ggplot2).

## 4. Conclusions

The combination of UVA and MVA results provided strong evidence to support the view that changes in the lipid composition of the growing media in *M. furfur* CBS 1878 produce qualitative and semi-quantitative differences in their volatiles profiles. Additionally, the growth phase also affects the type and intensity of the released VOCs, despite the yeast growth not being equal in the tested media. In the UVA and MVA, several VOCs were differentiated as meaningful factors, including carbon dioxide, hexan-1-ol, pentyl acetate, isomer5 of methyldecane, dimethyl sulphide, undec-5-ene, isomer2 of methylundecane, isomer1 of methyldecane, and 2-methyltetrahydrofuran. The differences observed using in vitro conditions in a controlled environment suggest how the presence of saturated (as PA) or unsaturated (as OA) fatty acids in the skin changes the VOCs profile in one species of the most important fungi genus from human skin microbiota. This species is particularly relevant because it is the only one known to be able to survive in PA, the most abundant saturated fatty acid in human sebum. Some VOCs are produced only when OA, PA, or a combination of both is present, but the complexity of the physiological and biochemical changes produced by the stressful nutritional conditions in these three restricted media should be studied further. The mDixon medium, with a more complex lipid composition, promotes the most diverse chemical profile, whereas the PA medium restricts the chemiodiversity and abundance of the VOCs produced. Our results demonstrate how variable are the volatile profiles of micro-organisms grown in different media. This study’s findings are especially important for chemotaxonomic purposes or for studying the ecological function of scents. In the current research, the variation on media allowed us to describe VOCs not previously reported for *Malassezia* or even for fungi. The ecological significance of our findings deserves future in vivo research to analyze the variability of lipid composition from skins of different humans, and how these variations induce changes in the volatiles profiles released by *M. furfur*. These modifications on human bouquets will determine many ecological interactions where VOCs play an important role, such as microbe–microbe, microbe–host, and microbe–insect interactions. Another important direction of research is the comparison of the VOCs profiles when *M. furfur* behaves as a commensal versus pathogenic, with the aim of better understanding the virulence of this micro-organism or the beneficial effect and how it changes according to all of the dynamic complex transformations of the microbiota.

## Figures and Tables

**Table 1:** Volatiles profiles of *M. furfur* in four media and two growth phases.

				Treatment	De			Ds			Oe			Os			Pe			Ps			OPe			OPs		
Compound	Crit.	RI	RI Exp	Ret. Time	X	SD	N	X	SD	N	X	SD	N	X	SD	N	X	SD	N	X	SD	N	X	SD	N	X	SD	N
Carbon dioxide *	*a*			1.3	7.7	4.2	5	14.6	15.8	5	9.8	6.6	5	8.4	6.2	5	61.5	21.4	5	45.3	22.0	5	2.5	0.4	5	2.4	1.7	5
Pentane *	*a*	500		1.6				1.1		1							2.0	0.3	2				0.1	0.1	2			
Dimethyl sulphide *	*a*	526		1.7	0.1	0.1	2	0.3	0.1	5																		
2-methylpent-1-ene	*a*	588		2.0				0.2	0.2	3	0.3		1															
2-methylpropan-1-ol *	*a,b*	629	624	2.2	0.1		1	0.2	0.1	5																		
Butan-1-ol *	*a,b*	663	658	2.6	0.5	0.2	5	0.4	0.1	5	1.0	0.3	5	0.8	0.3	5							0.1	0.1	5	2.1	3.5	5
2-methyltetrahydrofuran	*a,b*	678	665	2.6																			0.1	0.1	5	0.3	0.0	5
Isomer of pentan-2-one	*a*		678	2.8				0.6	0.3	4																		
Pentan-2-one *	*a,b*	674	684	2.8	0.8	0.2	3	6.2	2.0	5	0.2	0.1	4	3.6	5.6	5												
Hept-2-ene	*a,b*	est: 725	702	3.0																			0.6	1.0	4	2.4		1
Hept-3-ene	*a,b*	730	713	3.2																			0.1	0.1	5			
2,5-dimethylhexane	*a*	est: 688	723	3.4													2.4	0.4	3									
3-methylbutan-1-ol *	*a,b*	746	730	3.5	0.9	0.2	5	3.0	0.5	5										11.9		1						
2-methylbutan-1-ol *	*a,b*	745	734	3.5	0.1	0.0	5	0.2	0.1	2																		
3-methylpentan-2-one *	*a,b*	752	748	3.8				0.1	0.0	4																		
Octane *	*a,b*	800	800	4.6	49.3	11.4	5	20.1	10.9	5	2.8	1.4	4	1.6	1.3	5	11.6	10.2	5	4.8		1	51.9	2.7	5	15.3	3.7	5
Oct-2-ene *	*a,b*	810	806	4.8																			0.2		1	0.2	0.1	3
2-Furanmethanol	*a,b*	858	852	5.9	0.3	0.0	2	0.4	0.2	5																		
Hexan-1-ol *	*a,b*	870	865	6.3	17.9	7.2	5	32.9	11.8	5	72.9	12.6	5	80.5	6.3	5							28.6	3.2	5	60.0	21.4	5
Nonane	*a,b*	900	900	7.2	19.7	7.2	5	15.6	8.1	5	0.8		1	0.7	0.3	2	9.3	1.3	5	11.9	10.6	5	14.4	1.0	5	14.5	12.8	5
Pentyl acetate *	*a,b*	est: 924	912	7.6																			0.1	0.0	5	0.2	0.1	5
Unknown bp: 56			925	8.0																			0.1	0.0	4	0.1	0.0	2
Unknown compound 1 with Nitrogen and C=O	*a*		955	9.1																			0.0	0.0	3			
Hept-6-en-1-ol	*a,b*	est: 950	959	9.3																			0.3	0.1	5	1.3	0.5	5
Unknown1 bp: 95			980	10.0	0.1	0.0	3	0.1	0.0	2																		
Decane	*a,b*	1000	1000	10.7							0.7	0.1	3															
Dec-4-ene	*a,b*	est: 1023	1005	10.9																						0.3	0.0	3
Isomer1 of methyldecane	*a*	1001	1038	12.3	0.5	0.2	5	0.4	0.1	5																		
2,6,7-trimethyldecane	*a,b*	est: 1048	1052	12.9				1.9	0.4	5																		
Isomer4 of methyldecane	*a*		1059	13.2	0.3	0.1	3	0.2	0.0	3																		
Isomer5 of methyldecane	*a*		1069	13.6	0.5	0.4	5	0.5	0.1	5																		
(5-Ethyl-cyclopent-1-enyl)-methanol	*a,b*	1073	1073	13.7							0.8	0.4	3	1.2		1												
Isomer1 of Undec-1-ene	*a*		1079	14.0	1.9	0.9	3	1.1	0.1	3																		
Isomer2 of Undec-1-ene	*a*		1083	14.2							8.4	3.8	3	2.0	1.2	3												
Undec-5-ene	*a,b*	1092	1105	15.1																			0.3	0.0	5	0.9	0.2	5
Heptyl acetate	*a,b*	1110	1112	15.4																			0.1	0.0	3			
Isomer3 of Undec-1-ene	*a*		1114	15.5																						1.5	0.4	5
Isomer1 of methylundecane	*a*		1124	15.9													7.8	3.3	2									
Isomer2 of methylundecane	*a*		1124	15.9	0.4	0.2	5	0.4	0.0	5																		
Non-3-en-1-ol, (*Z*)- *	*a,b*	1158	1148	17.0							0.2		1													0.1	0.1	3
Non-2-en-1-ol, (*E*)- *	*a*	1120	1167	17.8																			0.3	0.2	4			
Unknown2 bp:95			1181	18.5				0.3	0.0	4																		
2-Nonyloxirane	*a,b*	est: 1205	1210	19.7																			0.2	0.0	4	0.4	0.1	5
4,7-dimethylundecane	*a,b*	est: 1185	1211	19.8				0.3	0.0	2							5.4	1.3	4	11.7	3.8	2						
Isomer2 of methyldodecane	*a*		1237	20.9							1.2	0.6	3	0.8		1												
Isomer3 of methyldodecane	*a*		1245	21.3	0.2	0.0	2				1.8	0.6	3	0.9		1							0.1	0.1	4	0.3	0.1	5
Isomer of Decenol	*a*		1268	22.3																			0.4	0.3	4			
Isomer5 of methyldodecane	*a*		1274	22.6							0.6	0.7	2															
Undecan-2-one *	*a,b*	1294	1292	23.4																16.7	8.7	4						
Isomer6 of methyldodecane	*a*		1295	23.5							1.0	0.0	2	1.5		1												
Isomer2 of methyldecanol	*a*		1295	23.5													4.7	2.2	2	12.2	2.0	2						
Unknown furanic	*a*		1301	23.7				0.7	0.5	2																		
Isomer1 of trimethyldodecane	*a*		1302	23.8							2.2	0.7	3	1.2	0.2	2												
Isomer2 of trimethyldodecane	*a*		1320	24.6							1.0	0.2	3	0.7		1												
Isomer of methylundecanol	*a*		1323	24.7							1.8	0.5	3	1.0		1												
Isomer1 of methyltridecane	*a*		1336	25.3							0.5	0.2	4	1.6	1.2	3												
Isomer2 of methyltridecane	*a*		1343	25.6							1.2	0.4	3	0.8		1												
Isomer3 of methyltridecane	*a*		1354	26.0							0.8	0.0	2															
Unknown3 bp:95			1381	27.2				0.3	0.1	4																		
Isomer of methyltetradecane	*a*		1496	31.9							1.2	0.4	3															
γ-Dodecalactone	*a,b*	1682	1679	37.5	0.3	0.2	3	0.4	0.1	4	0.6		1	0.9	0.1	3												

Average area percentage for each sample is represented as X, standard deviation is represented as SD, and number of replicates in which a volatile organic compound (VOC) was found as N. RI = Retention index published in literature. RI Exp. = Retention index relative to n-alkanes (C_8_ − C_20_) on a BP-5 column (30 m × 0.25 mm × 0.25 µm). We used two annotation criteria: *a* = Comparison of experimental mass spectra with the NIST14 database and *b* = experimental RI deviations below 30 units from the theoretical RI. Compounds with * represent VOCs reported previously for other Fungi in the mVOC 2.0 Database. In the table, only the isomers annotated and absent in the medium control analysis were reported (i.e., released by *M. furfur*).

## Supplementary Materials

The following are available online, Figure S1: Growth curves of *M. furfur* in the mDixon, oleic acid (OA), palmitic acid (PA), and OA+PA medium and their confidence intervals of 99% using a loess method. Figure S2: Animation of rotational distribution of data in the PCA analysis of the volatiles profiles of *M. furfur* growing in eight different experimental treatments. Table S1: Significant statistical univariate parameters found on 17 VOCs from *M. furfur* and compounds highlighted by PCA and PLS-DA. Table S2: Chemical composition of mDixon medium. Table S3: Chemical composition of oleic acid (OA), palmitic acid (PA), and OA+PA media. The components of the minimal medium (MM) are listed in Materials and Methods.
