# Supplementary material for: Yeast Smell Like What They Eat: Analysis of Volatile Organic Compounds of Malassezia furfur in Growth Media Supplemented with Different Lipids
Source: Molecules. 2019 Jan 24;24(3):419. doi: 10.3390/molecules24030419 (PMC6384859; doi:10.3390/molecules24030419)
Supplement: Supplementary file 1 [file molecules-24-00419-s001.zip › molecules-416385-supplementary-revised - original/3-SI.pdf]

Article

Yeast smell like what they eat: Analysis of Volatile Organic Compounds of *Malassezia furfur* in growth media supplemented with different lipids

Mabel González[1], Adriana M. Celis[2,3*], Marcela I. Guevara-Suarez[2,3], Jorge Molina[4] and Chiara Carazzone[1*]

[1] Laboratory of Advanced Analytical Techniques in Natural Products (LATNAP), Universidad de los Andes, Cra 1 No. 18A-12, Bogotá, ZIP Code 111711, Cundinamarca, Colombia; mabel.c.gonzalez@uniandes.edu.co (M.G.); c.carazzone@uniandes.edu.co (C.C.)

[2] Grupo de Investigación Celular de Microorganismos Patógenos (CeMoP), Universidad de los Andes, Cra 1 No. 18A-12, Bogotá, ZIP Code 111711, Cundinamarca, Colombia; acelis@uniandes.edu.co (A.C.); mi.guevara34@uniandes.edu.co (M.I.G.)

[3] Laboratorio de Micología y Fitopatología (LAMFU), Universidad de los Andes, Cra 1 No. 18A-12, Bogotá, ZIP Code 111711, Cundinamarca, Colombia; acelis@uniandes.edu.co (A.C.); mi.guevara34@uniandes.edu.co (M.I.G.)

[4] Centro de Investigaciones en Microbiología y Parasitología Tropical (CIMPAT), Universidad de los Andes, Cra 1 No. 18A-12, Bogotá, ZIP Code 111711, Cundinamarca, Colombia; jmolina@uniandes.edu.co (J.M.)

* Correspondence: c.carazzone@uniandes.edu.co; Tel.: +57-13394949 Ext. 1480. acelis@uniandes.edu.co (A.C.); Tel.: +57-13394949 Ext. 3757

Received: date; Accepted: date; Published: date

SUPPORTING INFORMATION

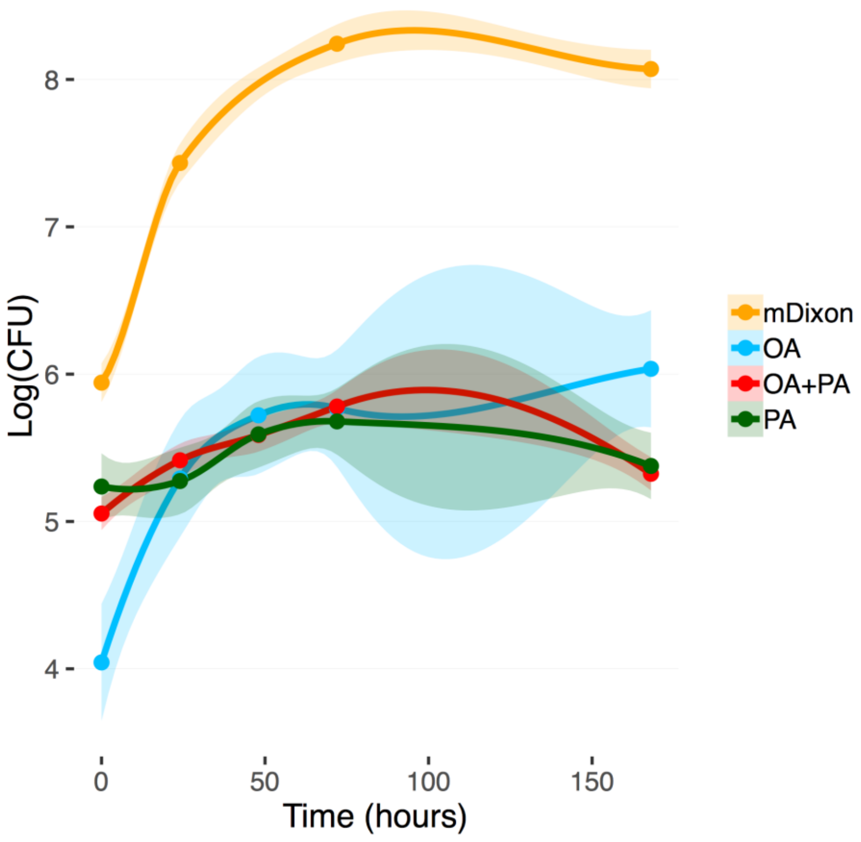

Figure S1. Growth curves of *M. furfur* in mDixon, oleic acid (OA), palmitic acid (PA) and OA+PA medium and their confidence intervals of 99% using a loess method.

3-FigureS2.gif

Figure S2. Animation of rotational distribution of data in the PCA analysis of the volatiles' profiles of *M. furfur* growing in eight different experimental treatments.

Table S1. Significant statistical univariate parameters found on 17 VOCs from *M. furfur* and compounds highlighted by PCA and PLS-DA.

	Statistics from ANOVA		Statistics from Kruskal-Wallis		PCA	PLS-DA
**Compound**	**F**	**pval**	**K**	**pval**		
Carbon dioxide	16.1	< 0.001	25.8	< 0.001	✓	✓
Octane	36.8	< 0.001	33.8	< 0.001		✓
~~1-~~Hexan-1-ol	92.5	< 0.001	34.6	< 0.001	✓	✓
Nonane	13.5	< 0.001	26.7	< 0.001		
~~2-~~Pentan-2-one	13.1	< 0.001	25.7	< 0.001		
3-methyl ~~-1-~~butan-1-ol	6.9	< 0.001	20.3	0.0049		
~~1-~~Butan-1-ol	7.2	< 0.001	32.8	< 0.001		✓
~~6-~~Hept-6-en-1-ol	106.8	< 0.001	22.4	0.0022		✓
~~*cis*-2-Nonene~~Pentyl acetate	132.1	< 0.001	22.4	0.0022	✓	✓
Isomer5 of methyldecane	56.2	< 0.001	22.0	0.0025	✓	✓
Dimethyl sulfide	29.9	< 0.001	14.3	0.0450	✓	✓
~~5-~~Undec-5-ene	371.0	< 0.001	22.4	0.0022	✓	✓
Isomer2 of methylundecane	94.5	< 0.001	22.0	0.0026	✓	✓
Isomer1 of methyldecane	133.7	< 0.001	22.0	0.0026	✓	✓
~~1,2-Epoxyundecane~~2-Nonyloxirane	51.0	< 0.001	19.0	0.0083		✓
Isomer3 of methyldodecane	4.0	0.0030	15.9	0.0258		
2-methyl-tetrahydrofuran	116.2	< 0.001	22.2	0.0023	✓	✓

Formatted: Font: Palatino Linotype, 10 pt

Formatted: List Paragraph, Bulleted + Level: 1 + Aligned at: 0.63 cm + Indent at: 1.27 cm

Table S2. Chemical composition of mDixon medium.

Component	Description or molecular structure	Percentage
malt extract		
	Bile composition (water 92 g/dL, bile salts 6 g/dL, bilirubin 0.3 g/dL, cholesterol 0.3 to 0.9 g/dL, FA 0.3 to 1.2 g/dL, lecithin 0.3 g/dL and 200 meq/L inorganic salts [1]	3.6
desiccated oxbile	Lecithins: phospholipids, glycolipids or triglyceride. Glycerophospholipids as phosphatidylcholine, phosphatidylethanolamine, phosphatidylinositol, phosphatidylse rine, and phosphatidic acid [2]	2.0
peptone	Natural sources of amino-acids, peptides and proteins	0.6
glycerol	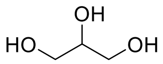 glycerol	0.2
oleic acid	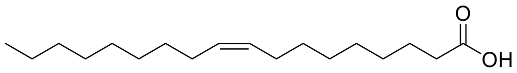 oleic acid	0.2
Tween 40	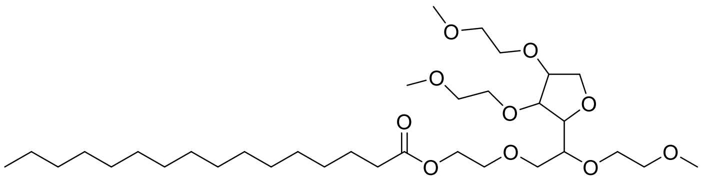 2-(2-(3,4-bis(2-methoxyethoxy)tetrahydrofuran-2-yl)-2-(2-methoxyethoxy)ethoxy)ethyl palmitate	1

Table S3. Chemical composition of oleic acid (OA), palmitic acid (PA) and OA+PA media. The components of the minimal medium (MM) are listed in Materials and Methods.

Medium	Components	Components found in the GC-FID analysis[3] or molecular structure	Percentage
OA	1% of Oleic acid	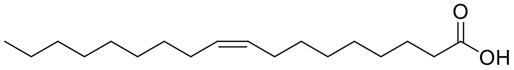 oleic acid	78%
		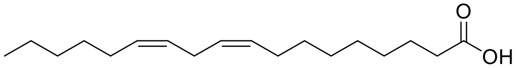 linoleic acid	10%
		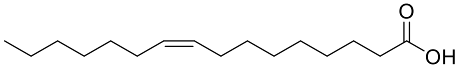 palmitoleic acid	3%
		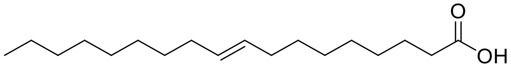 elaidic acid	2%
		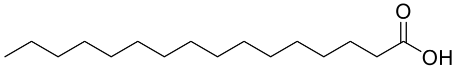 palmitic acid	6%
		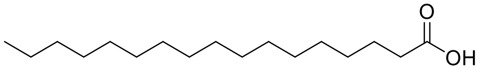 heptadecanoic acid	1%

	1% Tween 40	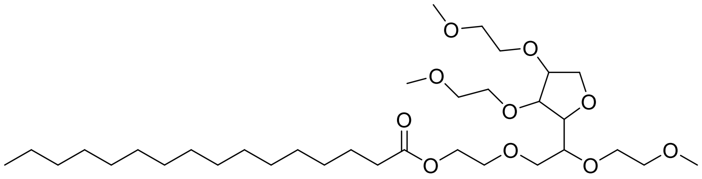<br>2-(2-(3,4-bis(2-methoxyethoxy)tetrahydrofuran-2-yl)-2-(2-methoxyethoxy)ethoxy)ethyl palmitate
	MM	
OA+PA	1% of a mixture 50:50 of Oleic acid and Palmitic acid	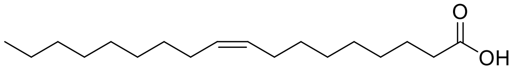<br>oleic acid<br>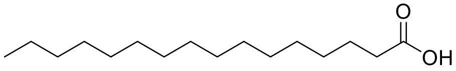<br>palmitic acid
	1% Tween 40	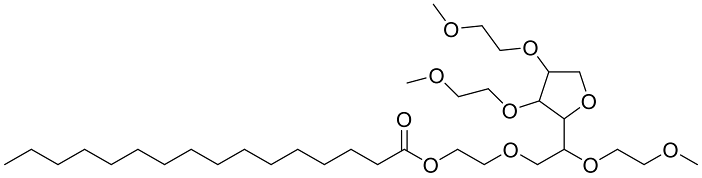<br>2-(2-(3,4-bis(2-methoxyethoxy)tetrahydrofuran-2-yl)-2-(2-methoxyethoxy)ethoxy)ethyl palmitate
	1% Brij-58	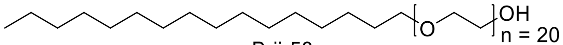<br>Brij-58
	MM	
PA	1% of Palmitic acid	

	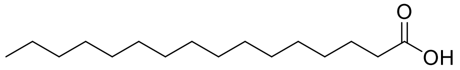<br>palmitic acid	98%
	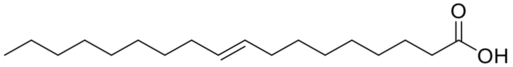<br>elaidic acid	2%
	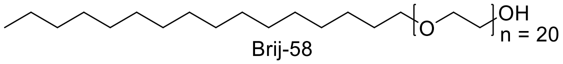<br>Brij-58	
1% Tween 40	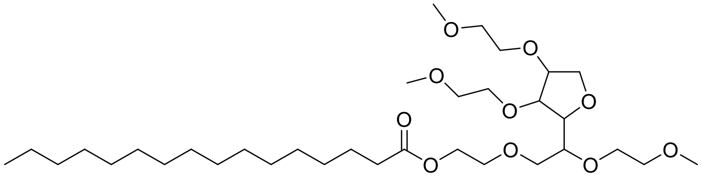<br>2-(2-(3,4-bis(2-methoxyethoxy)tetrahydrofuran-2-yl)-2-(2-methoxyethoxy)ethoxy)ethyl palmitate	
1% Brij-58	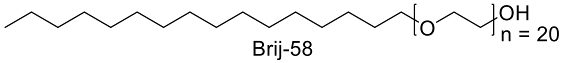<br>Brij-58	
MM		

References

1. Chopra, T.; Gokhale, R.S. *Chapter 12 Polyketide versatility in the biosynthesis of complex mycobacterial cell wall lipids*; 1st ed.; Elsevier Inc., 2009; Vol. 459; ISBN 9780123745910.

2. Celis, A.M. Unraveling lipid metabolism in lipid-dependent pathogenic Malassezia yeasts. Ph.D. Thesis, Utrecht University, 2017.

3. Triana, S.; de Cock, H.; Ohm, R.A.; Danies, G.; Wösten, H.A.B.; Restrepo, S.; González Barrios, A.F.; Celis, A. Lipid metabolic versatility in Malassezia spp. yeasts studied through metabolic modeling. *Front. Microbiol.* **2017**, *8*, 1–18, doi:10.3389/fmicb.2017.01772.
