# Supplementary figures and images for: Yeast Smell Like What They Eat: Analysis of Volatile Organic Compounds of Malassezia furfur in Growth Media Supplemented with Different Lipids
Source: Molecules. 2019 Jan 24;24(3):419. doi: 10.3390/molecules24030419 (PMC6384859; doi:10.3390/molecules24030419)

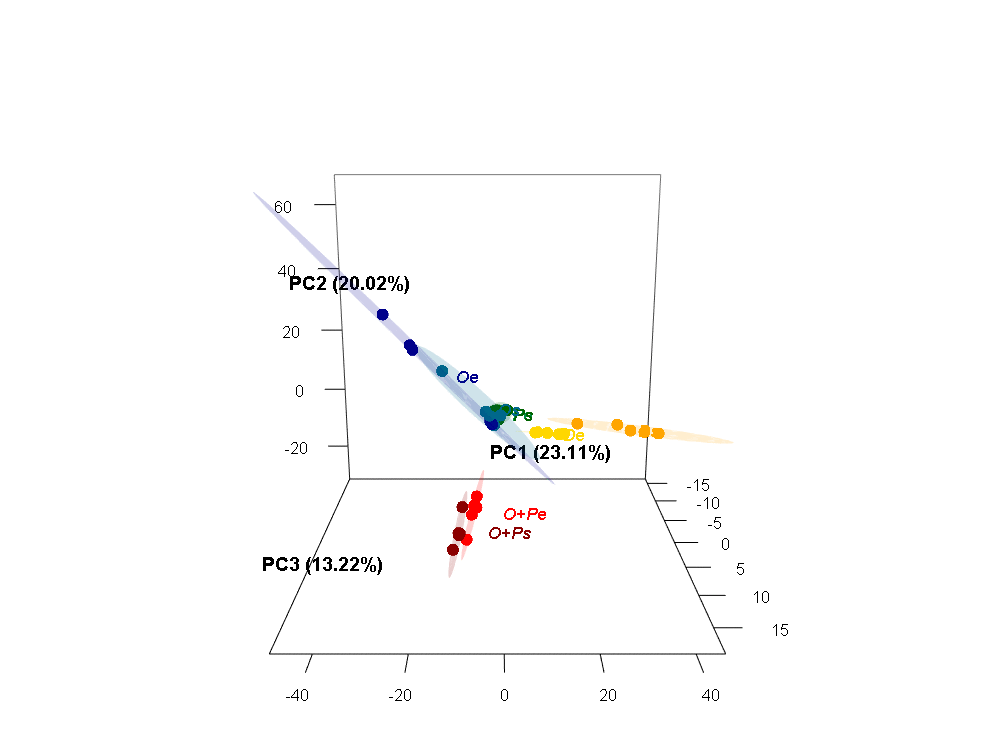

Supplement: Supplementary file 1 [file molecules-24-00419-s001.zip › molecules-416385-supplementary-revised - original/3-FigureS2.gif]

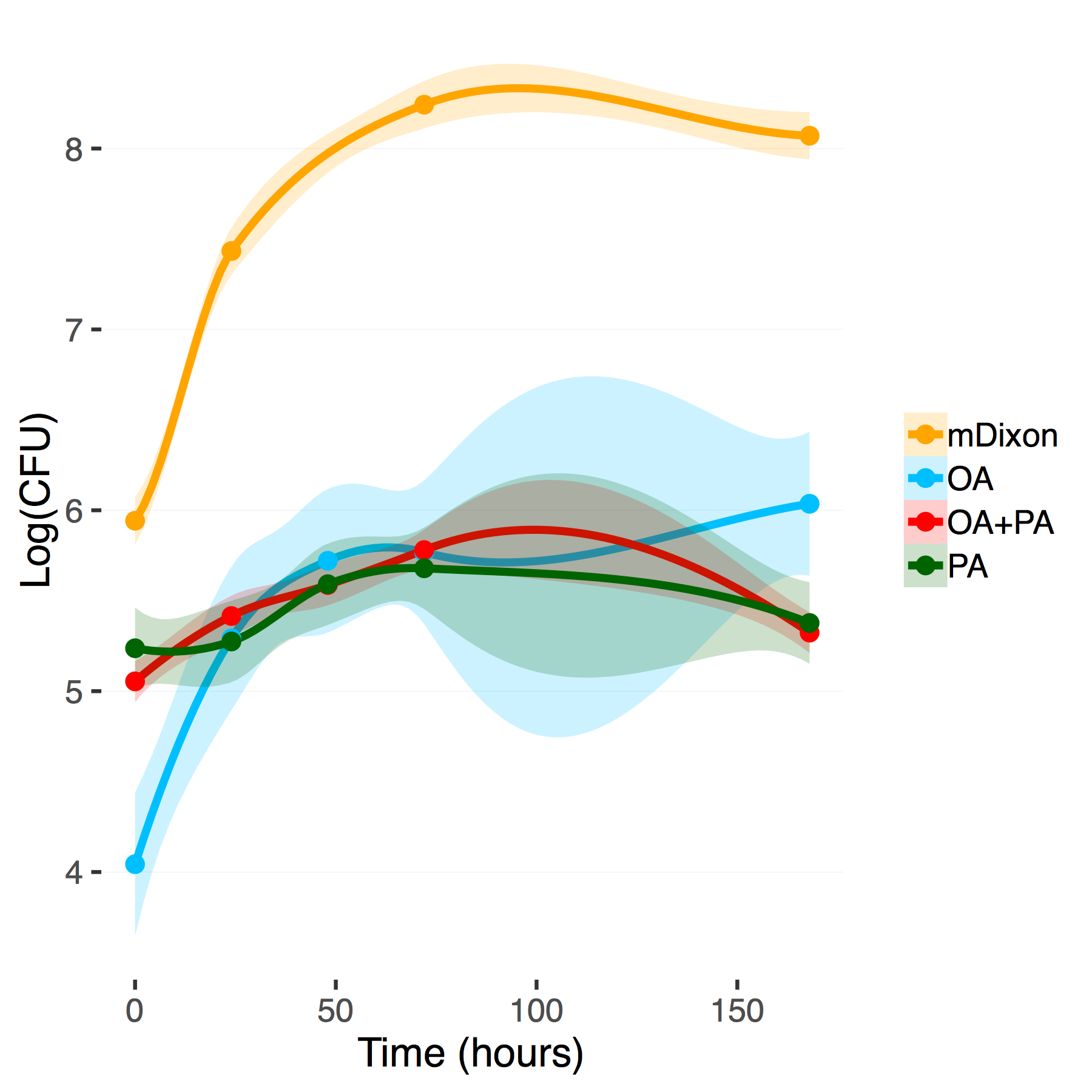

Supplement: Supplementary file 1 [file molecules-24-00419-s001.zip › molecules-416385-supplementary-revised - original/FigureS1-ic.tiff]
